# The Position of Circulating Tumor DNA in the Clinical Management of Colorectal Cancer

**DOI:** 10.3390/cancers15041284

**Published:** 2023-02-17

**Authors:** Ana Regina de Abreu, Ken Op de Beeck, Pierre Laurent-Puig, Valerie Taly, Leonor Benhaim

**Affiliations:** 1 Center of Medical Genetics, University of Antwerp and Antwerp University Hospital, Prins Boudewijnlaan 43, 2650 Edegem, Belgium; 2 Center for Oncological Research, University of Antwerp and Antwerp University Hospital, Wilrijkstraat 10, 2650 Edegem, Belgium; 3 UMR-S1138, CNRS SNC5096, Équipe labélisée Ligue Nationale Contre le Cancer, Centre de Recherche des Cordeliers, Université de Paris, 75006 Paris, France; 4 Department of Visceral and Surgical Oncology, Gustave Roussy, Cancer Campus, 114 rue Edouard Vaillant, 94805 Villejuif, France

**Keywords:** colorectal cancer, circulating tumor DNA, treatment, management

## Abstract

**Simple Summary:**

Currently, methods including endoscopy, radiology, and carcinoembryonic antigen levels allow for the detection of colorectal cancer (CRC) at an early stage and the ability to follow the evolution of the disease during treatment. However, these are not always sensitive and specific enough for timely intervention. This leads, amongst other consequences, to delays in treatment or even to overtreatment. Circulating tumor DNA (ctDNA) has shown promise in filling this gap, allowing treatment to be personalized at each stage of the disease and, thus, tailored to each patient’s needs. This review article focuses on the current clinical use and future direction of ctDNA for CRC management.

**Abstract:**

Colorectal cancer (CRC) is the third most common cancer type worldwide, with over 1.9 million new cases and 935,000 related deaths in 2020. Within the next decade, the incidence of CRC is estimated to increase by 60% and the mortality by 80%. One of the underlying causes of poor prognosis is late detection, with 60 to 70% of the diagnoses occurring at advanced stages. Circulating cell-free DNA (ccfDNA) is probably the most promising tool for screening, diagnosis, prediction of therapeutic response, and prognosis. More specifically, the analysis of the tumor fraction within the ccfDNA (circulating tumor DNA, ctDNA) has great potential to improve the management of CRC. The present review provides an up-to-date and comprehensive overview of the various aspects related to ctDNA detection in CRC.

## 1. Circulating Tumor DNA: Detection

Circulating cell-free DNA (ccfDNA) was first described in human plasma by Mandel and Métais in 1948 [1] and has been found to originate from various cell types, including cancer cells (ctDNA). Increased ccfDNA concentrations have been observed in situations of cell lysis and turnover, including pregnancy, intensive exercise, inflammation, infection, autoimmune diseases, diabetes, or soft-tissue injury [2,3,4,5]. The first connection between ccfDNA and cancer was made in 1977 by Leon et al. through the observation that ccfDNA concentrations were also increased in various types of cancers [6].

The above finding is also reflected in colorectal cancer (CRC). The rate of circulating tumor DNA (ctDNA) detection is highly variable across studies and ranges between 40 and 100% for localized tumors to nearly 100% in metastatic CRC [7,8]. ctDNA comprises a fraction of the total DNA circulating freely in the bloodstream and its proportion within ccfDNA depends on the cancer stage and ranges from 0.01 to 0.0001% to more than 50% [9]. Because of the absence of guidelines regarding ctDNA analysis, the interpretation and comparison of clinical studies first require a complete understanding of the detection process and potential issues.

The release of ctDNA depends on tumor burden, vascularity, and location, however, the detection of ctDNA is impaired at early stages. In patients with non-metastatic CRC, the ctDNA concentration is low, and its dilution among total ccfDNA may hinder its detection. Ideally, using the most sensitive and specific technique for ctDNA detection is preferable. Nevertheless, the choice of the detection method is also pragmatic, and the cost of ctDNA analysis needs to be acceptable enough to be translated into clinical practice. Another parameter is the processing time, specifically when the goal is to drive treatment decisions based on the ctDNA detection results. Moreover, there remain considerable differences in the (pre)-analytical conditions between published studies, which limits proper comparisons of ctDNA among individuals.

### 1.1. Pre-Analytical Conditions

The pre-analytical conditions refer to any variable encountered prior to sample analysis, such as the type of blood collection tubes, the centrifugation delay, the centrifugation protocols, the DNA isolation methods, and the storage conditions. These pre-analytical conditions are of particular importance to protect the integrity of the ccfDNA before downstream analysis [10,11,12]. A general finding is that delaying centrifugation from blood collection increases ccfDNA levels [13]. For example, the rapid lysis of white blood cells (WBCs) in the lavender top EDTA tubes results in genomic DNA (gDNA) release, which dilutes both the ccfDNA and its ctDNA fraction and alters the relative proportion. It is therefore necessary to have a fast plasma separation process (within 4 h). In contrast, other options are “blood preservative tubes” which have been developed to maintain blood cell integrity under various storage and shipping conditions [14].

Hence, controlling the pre-analytical variables and defining the best practices is of the utmost importance for reliable ctDNA analyses and a prerequisite for implementation into routine practice [15]. To the best of our knowledge, no official guidelines exist for the pre-analytical procedures of liquid biopsies, although efforts have been made to standardize these procedures [16]. Moreover, pre-analytical procedures are not routinely documented in published studies, which makes comparisons challenging.

### 1.2. Analytical Conditions

An overview of the main analytical methods is presented in Table 1. The current techniques can be broadly divided into two groups. The first approach consists of the targeted detection of tumor-specific alterations within ccfDNA. The analysis and detection of alterations within the primary tumor are an absolute prerequisite. Sensitivity and specificity are high, and the number of genes to be assessed simultaneously is limited. One of the advantages of this approach is that false positive results are less likely. These targeted approaches can be easily performed by PCR, real-time quantitative PCR (qPCR), digital PCR (dPCR), and droplet digital PCR (ddPCR). A classic example is the detection of *RAS* mutations that are present in about 50% of CRC patients and localized in hotspot regions that are easily targeted with a limited set of probes [17,18].

An alternative option consists of non-targeted (agnostic) methods. Indeed, it is possible to fish for alterations that frequently occur in CRC without baseline tumor tissue analysis. A non-targeted approach implies the use of optimized methods able to screen a large number of alterations concomitantly. Recent dPCR techniques (including droplet-based and microfabricated compartment-based platforms) can also be used, allowing high multiplexing through two to six-color detection [19,20,21]). For example, this technique can be used for methylation assays [22]. Optimized NGS methods including Safe-Sequencing System (Safe-SeqS), CAncer Personalized Profiling by deep Sequencing (CAPP-Seq), integrated Digital Error Suppression-enhanced CAPP-seq (iDES-enhanced CAPP-seq), and Base-Position Error Rate (BPER) have a high sensitivity. Nevertheless, they are more expensive and time-consuming than dPCR [23,24,25,26]. Non-targeted approaches have also been developed using Whole Exome Sequencing (WES) [27] and Whole Genome Sequencing (WGS) [28], allowing a complete genotyping and detection of de novo mutations but with a lower sensitivity and higher cost. In non-targeted approaches, the absence of detection could mean that the alterations were either absent in the blood or in the tumor itself [3].

Overall, detection methods must be selected according to the sampling conditions and the purpose of the studies [29]. For clinical applications, the most favorable test is time- and cost-effective with acceptable sensitivity and specificity.

### 1.3. Delay of Sampling

#### Delay between Surgery and ctDNA Sampling

The best timing for ctDNA sampling is controversial, and only a few publications have been dedicated to this topic. In non-metastatic CRC, a drop in ccfDNA concentration immediately after surgical treatment has been reported [30]. The ccfDNA rises beginning at 24 h after surgery and can be used to discriminate patients with recurrence after 48 h [30]. However, early blood collection (before week 4) may theoretically reduce the sensitivity of ctDNA detection because of ccfDNA release as a consequence of the surgical trauma [31]. In the study by Scholer et al., blood samples were collected on day 8, day 30, and every month. Interestingly, out of 26 operated patients, 2 were ctDNA+ eight days after surgery, and 2 others became ctDNA+ one month after surgery [32]. Overall, collecting blood early after surgery might be more relevant for immediate clinical application but must be weighed against a higher rate of false negatives.

## 2. ctDNA: Clinical Applications

**Figure 1:**
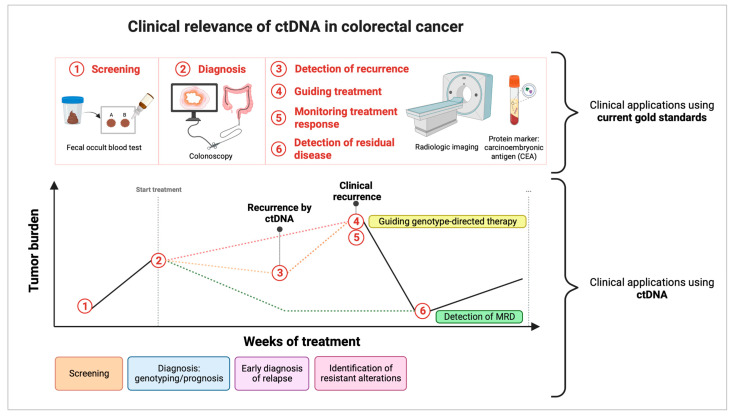
Clinical relevance of ctDNA in colorectal cancer. This figure depicts the critical applications in the clinical setting using both the current clinical gold standards and ctDNA. These include tumor genotyping in cancer diagnosis, assessing treatment response, tracking minimal residual disease and relapse, and monitoring clonal evolution. (1) Screening is routinely performed to detect CRC at an early stage using a fecal occult blood test (FOBT), but replacement by ctDNA has yet to be encouraged. (2) Diagnosis is performed before therapy to confirm the tumor’s presence. When using ctDNA, genotyping could determine the tumor profile and identify patients with a high tumor burden. Determination of tumor burden has the potential to help guide neo-adjuvant or adjuvant therapy and monitor response. (3/6) Detection of residual disease and recurrence is performed using radiologic imaging and carcinoembryonic antigen (CEA) detection. However, the former suffers from a delay in detection and the latter suffers from a lack of sensitivity. Assessment of ctDNA after therapy facilitates the detection of both emerging resistance mutations and minimal residual disease (MRD) before progression, with the potential for the non-invasive prediction of recurrence. (4/5) Guiding treatment and monitoring treatment response occurs based on the presence or absence of tumor lesions. On the other hand, ctDNA can guide genotype-directed therapy and allows for the monitoring of the response to treatment based on tumor burden. When acquired resistance to targeted therapies occurs, ctDNA can detect specific mechanisms or resistance, considering the different clones present within the primary tumor and all metastatic sites, and can guide treatment adjustments. In contrast, imaging and the CEA marker can detect the emergence of resistance without knowing the mechanisms of resistance. Created with BioRender.com.

Figure 1 illustrates some applications of ctDNA monitoring before and after treatment in various settings. This section will describe the significance of ctDNA as a screening, diagnostic, prognostic, predictive, minimal residual disease, and recurrence marker.

### 2.1. ctDNA for Early Cancer Detection: Screening

#### Screening

Because survival is highly affected by the stage at diagnosis, early detection of CRC is critical [33]. Most screening programs for CRC are currently based on a non-invasive stool-based test, either the guaiac-based fecal occult blood test (gFOBT) or the immunological fecal occult blood test (iFOBT), also referred to as the fecal immunochemical test (FIT). These tests are not specific and present a low sensitivity for the detection of CRC. When positive, CRC must be confirmed by a complete colonoscopy, which is invasive, expensive, and often requires sedation [33,34,35,36].

To circumvent these limitations, ctDNA has been explored as a potential screening tool for CRC. Table 2 shows a comparison of the different CRC screening methods. Taking advantage of the fact that aberrant DNA methylation is generally one of the first steps in CRC carcinogenesis, several methylation signatures have been explored [37] using one [38] or multiple gene methylation profiles [39,40]. Amongst various methods, the best sensitivity (75–81%) and specificity (96–99%) so far have been provided by the Epi ProColon^®^ 2.0 test (Epigenomics AG, Berlin, Germany) which is based on the detection of hypermethylation on the *SEPT9* promoter [38]. To date, this test is the only blood-based qualitative screening test accepted by the FDA for CRC. In a population of 7941 asymptomatic individuals (PRESEPT), the SEPT9 detected CRC with sensitivities of 35.0%, 63.0%, 46.0%, and 77.4%, in stages I to IV, respectively. In this population, the sensitivity for advanced adenomas was only 11.2% [41] which remains insufficient to replace standard colonoscopy screening. However, one of the main advantages of the *SEPT9* test is the increased patient compliance compared to the colonoscopy [42,43]. The Epi ProColon^®^ 2.0 test aims explicitly at detecting CRC, but early detection tests are being developed that target a variety of cancers, including CRC. GRAIL Inc. recently published a novel test (Galleri^®^ test) for the early detection of more than 50 types of cancer simultaneously, including CRC. This test analyses specific methylation patterns in ccfDNA that have been associated with many cancer entities. The tissue of origin can be predicted with 96% specificity and 93% accuracy. Moreover, the sensitivity in all cancer types was 18% (stage I), 43% (stage II), 81% (stage III), and 93% in stage IV. This corresponds to the sensitivity in detecting CRC, approximately 28% (stage I), 70% (stage II), 78% (stage III), and 97% (stage IV). These results indicate an important potential benefit to using methylated ccfDNA for cancer detection [44].

In general, the concentration of ctDNA in patients’ blood at early stages is low or nonexistent, making its detection challenging for screening purposes. The American Society of Clinical Oncology (ASCO) and the College of American Pathologists (CAP) has concluded that there is still little evidence for the clinical validity of ctDNA detection for cancer screening [46]. However, ctDNA detection could be combined with traditional screening methods to improve the diagnosis of CRC at an early stage [47,48,49].

### 2.2. The Value of ctDNA Detection at Diagnosis

At diagnosis, the frequency of ctDNA detection is around 10–50% for stage I, 20–89% for stage II, 30–90% for stage III, and 60–100% for stage IV [8,32,50,51,52,53,54,55] (see Table 3).

In the non-metastatic setting, the prognostic value of ctDNA detection at baseline (i.e., before surgery) is unclear. In the study of Reinert et al., almost all patients (stage I-III) presented with ctDNA+ detection before surgery, which was not associated with the risk of recurrence [8]. In the ALGECOLS (Presence of Circulating Tumour DNA in Colorectal Cancer) study (NCT01198743), 27.5% of the patients were ctDNA+ before surgery. These ctDNA+ patients showed a higher rate of recurrence (32.7% versus 11.6% in ctDNA− patients, *p* = 0.001). In addition, the time to recurrence (TTR) was significantly shorter in ctDNA+ patients compared to ctDNA− patients (adjusted HR = 3.58, 95% CI 1.71–7.47) [51]. These observations were similar in a large Chinese cohort of 276 patients with stage II/III tumors. Pre-operative ctDNA+ patients showed a reduced RFS compared to pre-operative ctDNA− patients (HR 5.66; 95% CI 1.72–18.57; *p* = 0.004) [56]. Those discrepancies suggest that survival is probably associated with ctDNA concentration rather than a simple ‘yes or no’ detection.

In patients with metastatic CRC, the tumor load before treatment is an important prognostic factor [58,59,60]. The ctDNA concentration before chemotherapy administration can be considered a continuous variable, with the highest concentrations being associated with the shortest survival [61].

### 2.3. ctDNA as a Prognostic Biomarker in CRC Stage I–III: Detection of Minimal Residual Disease

After curative-intent surgery, adjuvant chemotherapy (ACT) is routinely delivered to patients with high-risk stage II or stage III CRC. However, >50% of stage III and >80% of stage II patients are exposed to unnecessary chemotherapy. In fact, the 5-year DFS rate of stage II and low-risk stage III patients who underwent surgery alone has been reported as 78–91% and 78%, respectively [62]. Since the treatment is associated with lifetime side effects (e.g., chemotherapy-induced neuropathy), one aims to reduce the incidence of ACT when proven unnecessary [63].

#### 2.3.1. Detection of MRD/Recurrence after Surgery

In a pioneering study of 230 patients with stage II colon cancer, Tie et al. investigated the ability to identify patients at high risk of recurrence by detecting post-operative ctDNA [64]. The delay for post-operative plasma withdrawal was 4 to 10 weeks. The rate of ctDNA-positive detection was 8.7% for the whole cohort. In patients not treated with ACT (*n* = 178), ctDNA was detected post-operatively in 7.9% and was associated with a 79% recurrence at a median follow-up time of 27 months. The recurrence occurred in only 9.8% of patients with negative ctDNA (HR 18; 95% CI, 7.9 to 40; *p* < 0.001). In this study, 52 patients with histological high-risk stage II CRC were treated with ACT. Among them, six were positive for ctDNA detection after surgery, and three recurred despite the adjuvant treatment [64].

Using a tumor-informed Safe-SeqS platform, Tie et al. further analyzed the ctDNA status in a cohort of patients with stage III CRC [50]. The ctDNA was detectable in 21% of the cohort 4 to 10 weeks after surgery. A recurrence was observed in 42% of the patients with post-operative ctDNA+.

In a longitudinal cohort study, ctDNA was used to monitor tumor burden in 21 CRC patients (stages I-III) who underwent ctDNA analysis three months after complete surgery. In all six patients with detectable ctDNA, the recurrence occurred within three years. In patients without detectable ctDNA, the recurrence rate was 27% (4/15) {HR, 37.7; 95% CI, 4.2–335.5; *p* < 0.001} [32].

Mixing a population of patients with stage II and III, Li et al. found 27.8% disease progression for ctDNA-positive patients after surgery (ctDNA sampling within one week after surgery and follow-up of 6 months) compared to 4.4% for those who were ctDNA-negative (Fisher test, OR 7.9, *p* = 0.0169, 95% CI) [65].

Another study, including 125 patients with stages I-III, similarly showed that ctDNA-positive patients were seven times more likely to relapse after surgery than ctDNA-negative patients (HR, 7.2; 95% CI, 2.7–19.0; *p* < 0.001). Shortly after ACT, ctDNA-positive patients were 17 times more likely to relapse (HR, 17.5; 95% CI, 5.4–56.5; *p* < 0.001), and all seven patients who were ctDNA positive after ACT experienced relapse [8].

Taieb et al. also worked on the prognostic value of post-operative ctDNA in the IDEA-FRANCE trial (NCT00958737). Overall, 1017 patients were included, of which ctDNA samples were available post-surgery and pre-chemotherapy. Among them, 877 were ctDNA-negative (86.2%) and 140 ctDNA-positive (13.8%) after surgery. With a median follow-up of 6.6 years, the 3-year disease-free-survival (DFS) rate for ctDNA-positive and -negative patients was 66.39% and 76.71%, respectively (*p* = 0.015) [66].

Similarly, Benhaim et al. evaluated the pertinence of longitudinal detection and quantification of ctDNA prospectively as a prognostic marker of recurrence in the ALGECOLS (Presence of Circulating Tumor DNA in Colorectal Cancer) study (NCT01198743). The ctDNA analysis was performed before and after surgery in 184 patients (stage II-III) during 3–4 years of follow-up using ddPCR. After surgery, 18/171 (10.5%) patients were ctDNA+. Positive ctDNA levels after surgery were associated with a 44.4% recurrence rate versus 13.7% in ctDNA− patients (*p* = 0.003) [51].

Chen et al. also found that post-operative serial ctDNA detection predicted a high risk for recurrence. Low recurrence risk was observed in ctDNA− patients, with a 2-year RFS rate of 89.4% {95% CI 85.1–93.9%}, while ctDNA+ patients had an extremely high recurrence risk compared to ctDNA− patients {HR 10.98; 95% CI 5.31–22.72}, with a 2-year RFS rate of 39.3% {95% CI 21.5–71.8%}.

The abovementioned studies that have shown the relevance of ctDNA as a marker for the detection of MRD are listed below in Table 4.

#### 2.3.2. ctDNA Clearance after Treatment

At each stage of treatment, variations in ctDNA concentration and ctDNA clearance might reflect treatment efficacy. However, the rate of ctDNA clearance has not been evaluated because of the lack of extensive studies with longitudinal sampling. The main results are summarized in Table 5.

Rate of ctDNA clearance after surgery: Within patients with pre-operative ctDNA+, around 75–92% have ctDNA clearance after surgery.

- In stages I–III CRC, Reinert et al. observed that 84/94 (89.4%) patients became ctDNA negative and 10/94 (10.6%) patients became ctDNA positive after surgery.
- In stages II–III CRC, the ctDNA status changed from positive to negative in 75–92% of the patients after surgery [51,56].

Rate of ctDNA clearance after adjuvant chemotherapy: In stages I–III CRC, the rate of ctDNA clearance observed under chemotherapy was between 50% and 68%. This rate is approximately the same for stage II and stage III CRC.

- In stages I–III, Reinert et al. observed 30% of patients who cleared ctDNA after ACT and stayed disease free throughout the study [8].
- In stage II, Tie et al. observed that post-operative ctDNA+ remained negative after ACT in three out of six patients [64].
- In stage III, the ctDNA status changed from positive to negative in 50–68% of the patients after completion of chemotherapy treatment [50,51,67].

ctDNA clearance is associated with a superior RFS in most series: In stages II and III, superior RFS was observed when ctDNA became undetectable after chemotherapy (HR 5.11; *p* = 0.02) [64]. The absence of ctDNA clearance after chemotherapy is associated with a rate of 30% RFI at three years (HR, 6.8; 95% CI, 11.0–157.0; *p* < 0.001) [50].

#### 2.3.3. Value of ctDNA in the Prediction of Relapse before Conventional Imaging Techniques

Predicting relapse before radiologic recurrence is necessary. ctDNA detection can anticipate radiological recurrence with a lead time of 3 to 12 months, as described in recent publications (see Table 6). These results are clearly subject to bias, as the usual interval between plasma sampling is three months, whereas, for imaging assessments, it is six months. Although this anticipation is crucial, we should be aware that false-positive results exist and overall survival has not been shown to increase with the earlier treatment of relapses. The French trial CIRCULATE-MRD has recently been funded and will soon open for inclusion to address this question. In addition, the sensitivity and specificity of ctDNA detection during follow-up should be further studied to determine whether longitudinal sampling could be a way to avoid or delay (reduce) imaging follow-up.

### 2.4. ctDNA as a Quantitative Monitoring Tool in Predicting Response to Treatment (Stage IV)

#### 2.4.1. ctDNA Concentration during Treatment

The ctDNA concentration varies throughout the treatment duration; it reflects a response to treatment and allows for the selection of non-responding patients. Table 7 summarizes the main studies that have addressed this knowledge area.

Early changes in ctDNA concentration during the treatment course predict subsequent radiologic responses, suggesting that ctDNA is a marker of therapeutic efficacy [61,74,75]. Moreover, in some studies, changes in ctDNA also affected PFS and OS, where patients with relatively low ctDNA concentrations showed longer PFS and OS compared to patients with higher ctDNA concentrations [61].

Overall, the longitudinal surveillance of ctDNA allows for the early detection of relapse and response to intervention [32,76]. The detection of ctDNA could participate in the individual management of patients based on their tumor’s genetic profile, as the behavior of cancer in response to therapy can be predicted by determining ctDNA concentrations [29].

#### 2.4.2. ctDNA Predicts Response to Targeted Therapy

A prime example concerns the eligibility for anti-EGFR treatment, where the *RAS* mutation status of tumor tissue must be determined prior to formulating a treatment plan [17,18]. Since the concordance level in *KRAS* mutational status between tumor tissue and ctDNA is high (∼92%) [77], the detection of *KRAS* mutations in ctDNA has been proposed as a rapid and minimally-invasive alternative method to tissue biopsy for predicting the response to anti-EGFR treatment [78]. Interestingly, *KRAS* mutations have also been detected in ctDNA, although the primary tumor was considered wild-type. These circulating mutations may reflect the existence of minor cell subclones in the primary tumor or its related metastases [5]. Knebel et al. described the monitoring of a patient with *KRAS* wild-type mCRC treated with chemotherapy combined with anti-EGFR therapy [79]. Surprisingly, *KRAS* mutations in ctDNA were detected after the first exposure to anti-EGFR therapy but before clinical progression. Subsequently, the evolution of the disease went along with increasing concentrations of *KRAS*-mutated ctDNA. These results support the importance of the longitudinal monitoring of *KRAS* mutations in ctDNA before and during anti-EGFR therapy for the early detection of increasing cell clones that could be associated with drug resistance [79].

The emergence of *RAS* mutations in initially *RAS* wild-type tumors is a well-known mechanism of acquired resistance to anti-EGFR therapy. Nevertheless, whether these mutations are acquired de novo or whether initially undetectable mutant subclones proliferate through clonal selection and evolution remains unclear [80,81]. A subsequent treatment involving the withdrawal of EGFR blockade may be followed by an increase in the proportion of wild-type (sensitive) clones and a decrease in resistant (*RAS* mutant) clones, even to undetectable levels [81]. This work laid the foundation for the activity of anti-EGFR rechallenge. The CRICKET (Cetuximab Rechallenge in Irinotecan-Pre-treated mCRC, *KRAS*, *NRAS,* and *BRAF* wild-type Treated in 1st line With Anti-EGFR Therapy) trial (NCT02296203) demonstrated that a rechallenging strategy (in a third-line setting) with cetuximab and irinotecan can be effective, whereby evaluating the *RAS* mutation status on ctDNA might help select candidate patients and guide therapeutic decisions [71]. Patients with *RAS* wild-type ctDNA had a significantly longer PFS than those with *RAS* mutated ctDNA (median PFS: 4.0 vs. 1.9 months; hazard ratio: 0.44; 95% CI, 0.18–0.95; *p* = 0.03) [71].

Several preclinical studies have suggested that *ERBB2* (HER2) copy number gain is a negative predictor of response to anti-EGFR therapy [82]. Investigators of the HERACLES A study, a phase II trial of trastuzumab and lapatinib in chemotherapy and EGFR antibody-refractory HER2-positive mCRC patients, reported that ctDNA precisely predicted the response to anti-HER2 therapy in HER2-positive CRC [72]. In total, 47 of 48 samples from 29 patients had detectable ctDNA, and 46 out of 47 samples were HER2-positive {2.55–122 copies; 97.9% sensitivity (95% CI, 87.2–99.8%)}. These results support the use of adequately validated ctDNA testing as an alternative to tissue biopsy to identify individuals who may benefit from anti-HER2 therapy [72].

Herbst et al. suggested that detecting *HPP1* methylation in ctDNA could be used as an early marker to identify patients likely to benefit from a combination of chemotherapy and bevacizumab [73]. Before starting treatment, 337 of 467 patients had detectable methylated *HPP1* ctDNA. Two to three weeks after starting treatment, methylated *HPP1* ctDNA levels decreased to undetectable levels in 167 of 337 patients. These patients showed improved OS compared to patients with continued detection of methylated *HPP1* ctDNA. In addition, methylated *HPP1* ctDNA is predictive for combination therapy as early as 3 weeks after the start of treatment, whereas radiological imaging cannot do so until 12 or 24 weeks [73].

### 2.5. ctDNA as a Tool for Guiding Treatment

Table 8 lists several ongoing trials using ctDNA to guide treatment. In the non-metastatic setting, ways to better select adjuvant treatment are actively sought. For example, the ongoing ctDNA-guided single-arm phase II CHRONOS (Rechallenge With Panitumumab Driven by *RAS* Clonal-Mediated Dynamic of Resistance) trial (NCT03227926) aims to determine which patients are eligible for anti-EGFR rechallenge. This study uses the ctDNA analysis of *RAS*, *BRAF*, and *EGFR* mutations to drive anti-EGFR rechallenge therapy in mCRC [83]. Based on the same model, second-line rechallenge with cetuximab is under evaluation in the CRICKET trial [71].

In the metastatic setting, several studies have assessed the accuracy of ctDNA-based genotyping in selecting patients for mutation-directed therapy [84,85,86]. Currently, the ongoing prospective, multicentric interventional study (Following Therapy Response Through Liquid Biopsy in Metastatic Colorectal Cancer Patients, FOLICOLOR) in Belgium is evaluating the utility of ctDNA genotyping to monitor clinical response and guide therapeutic decision-making. In patients with unresectable metastatic disease, progressive disease is identified by *NPY* methylation in ctDNA. Two primary endpoints are (1) investigating whether ctDNA can detect progressive disease earlier than conventional monitoring based on CT imaging and (2) whether adapting treatment based on ctDNA could improve progression-free survival and overall survival.

**Table 8:** Ongoing clinical trials in CRC patients evaluating the use of ctDNA.

Name of Study and Country	Recruitment Status	Patient Population	Sample Size	ctDNA Detection Method	Intervention	Primary Objective
Cetuximab Rechallenge in irinotecan-pre-treated mCRC, KRAS, NRAS, and BRAF Wild-type Treated in 1st Line With Anti-EGFR Therapy (CRICKET) (NCT02296203) Italy [71]	Active, not recruiting	*KRAS, NRAS,* and *BRAF* wild-type, irinotecan-resistant mCRC patients who have progressed after an initial response to a first-line cetuximab-containing therapy	28	ddPCR	Cetuximab and irinotecan (single-arm trial)	Overall response rate
Rechallenge With Panitumumab Driven by RAS Dynamic of Resistance (CHRONOS) (NCT03227926) Italy [83]	Active, not recruiting	*RAS* wild-type mCRC patients who have progressed on first-line anti-EGFR therapy and whose *RAS* mutation load has decreased over 50% compared to the mutation load at the time of progression on first-line anti-EGFR therapy	129	ddPCR	Panitumumab monotherapy (single-arm trial)	Overall response rate
Circulating Tumor DNA Based Decision for Adjuvant Treatment in Stage II Colon Cancer based on ctDNA (CIRCULATE-PRODIGE 70-trial) (NCT04120701) France [87]	Recruiting	Stage II colon cancer patients who underwent curative-intent surgery	1980	ddPCR	ctDNA-positive randomized into two arms (1) Control arm: observation (2) Experimental arm: adjuvant mFOLFOX6 ctDNA-negative: surveillance	3-year disease-free survival
Following Therapy Response Through Liquid Biopsy in Metastatic Colorectal Cancer Patients (FOLICOLOR) Belgium	Recruiting	Unresectable, metastatic colorectal cancer patients receiving first-line treatment (pembrolizumab, panitumumab, or FOLFOX/FOLFIRI with(out) targeted therapy)	336	ddPCR	Control arm: Treatment decisions are guided by radiographic evaluation Experimental arm: Treatment decisions are guided by ctDNA	Primary: To determine the proportion of patients in which PD can be detected earlier in ctDNA than with conventional CT imaging Secondary: - PFS - 3-year overall survival
Circulating Tumour DNA Analysis Informing Adjuvant Chemotherapy in Stage II Colon Cancer (DYNAMIC) (ACTRN12615000381583) Australia [88]	Closed	Stage II colon cancer patients who underwent curative-intent surgery	455	Safe-SeqS	Control arm: All decisions were based on conventional clinicopathological criteria) Experimental arm: ctDNA informed (ctDNA positive: adjuvant chemotherapy; ctDNA negative: no adjuvant chemotherapy)	2-year recurrence-free survival
Circulating Tumor DNA Analysis Informing Adjuvant Chemotherapy in Stage III Colon Cancer (DYNAMIC-III) (ACTRN12617001566325) Australia [89]	Recruiting	Stage III colon cancer patients who underwent curative-intent surgery	1000	Safe-SeqS	Control arm: standard of care treatment Experimental arm: ctDNA informed (ctDNA negative: therapy de-escalation; ctDNA positive: therapy escalation)	3-year recurrence-free survival
Tracking Mutations in Cell Free Tumor DNA to Predict Relapse in Early Colorectal Cancer (TRACC) (NCT04050345), United Kingdom [90]	Recruiting	High-risk stage II and III patients with CRC who have measurable ctDNA pre-operatively and underwent R0 resection	1000	ddPCR	Control arm: standard of care ACT after surgery Experimental arm: ctDNA-guided ACT (ctDNA-negative: therapy de-escalation of ACT for 3 months with single Cape, or no chemotherapy; ctDNA positive: 3 months CapOx)	1. The incidence of pre-operatively detectable ctDNA in stage II and III CRC patients 2. The correlation between post-operatively detectable ctDNA and DFS
Circulating Tumor DNA Analysis to Optimize the Operative and Postoperative Treatment for Patients With Colorectal Cancer—Intervention Tial 2 (IMPROVE-IT2) (NCT04084249) Denmark [91]	Recruiting	Stage I and II patients with colon cancer who underwent surgery	254	ddPCR Targeted error correction sequencing (TEC-Seq) [52]	Control arm: surveillance according to current Danish Guidelines with CT-scans at 12- and 36-months post-operative and colonoscopy every 5 years until age 75 Experimental arm: ctDNA-guided surveillance every 4 months postoperatively. (1) ctDNA-positive: patients undergo a whole-body FDG-PET/CT scan and colonoscopy. (2) ctDNA-negative: high-intensive radiological surveillance with FDG-PET/CT-scan every 3 months until recurrence detection or 21 months have passed	Fraction of patients with relapse receiving curative-intended resection or local treatment
Circulating Tumor DNA Testing in Predicting Treatment for Patients With Stage IIA Colon Cancer After Surgery (COBRA) (NCT0406810 US [92]	Recruiting	Patients whose stage II colon cancer has been resected and who have no traditional high-risk features	1408	LUNAR™ (Guardant Health Inc.)	Control arm: Standard of care, observation Experimental arm: Prospective testing for ctDNA. (1) ctDNA-positive: treatment with 6 months of adjuvant (FOLFOX) chemotherapy (2) ctDNA-negative: active surveillance	1. Clearance of ctDNA with adjuvant chemotherapy 2. Recurrence-free survival for ctDNA-positive patients treated with or without adjuvant chemotherapy
Circulating tumor DNA-guided adaptive platform trials to refine adjuvant therapy for CRC (CIRCULATE-Japan, consists of the 3 trials (GALAXY, VEGA & ALTAIR)), Japan [93]						
Genetic Alterations and clinical record in radically resected colorectal cancer revealed by Liquid biopsy and whole eXome analYsis (GALAXY) (UMIN000039205)	Recruiting	Stage II high-risk and stage III low-risk CRC patients who have recurrence after initial registration and are eligible for radical surgical resection	2500	Signatera™ (Natera Inc.)	Observational study Based on ctDNA results in this study, patients can be enrolled in investigator-initiated phase III trials, either the VEGA (if ctDNA-negative) or the ALTAIL (if ctDNA-positive) trial (see below).	1. Disease-free survival 2. Sensitivity and specificity of ctDNA for the presence of lymph node metastases in additional colorectal resections
Study to Compare CAPOX Therapy as Post-operative Adjuvant Chemotherapy with Surgery Alone in Patients with Completely Resected Circulating Tumor DNA-negative High-risk Stage II and Low-risk Stage III Colon Cancer (VEGA) (jRCT1031200006)	Recruiting	Colon cancer patients that have a negative ctDNA status at week 4 after surgery in the GALAXY study	1240	Signatera™ (Natera Inc.)	Control arm: Observation Experimental arm: CapOx therapy for 3 months	Disease-free survival
Initial Attack on Latent Metastasis Using TAS-102 for ctDNA Identified Colorectal Cancer Patients After Curative Resection (ALTAIR) (NCT04457297)	Recruiting	CRC patients that have a positive ctDNA status within the previous 3 months at any time after surgery in the GALAXY study, and no obvious relapse on CT-scan	240	Signatera™ (Natera Inc.)	Control arm: Placebo Experimental arm: 6 months of oral trifluridine/tipiracil (FTD/TPI)	Disease-free survival

## 3. Rectal Cancer: The Current State of Management

Approximately one-third of all newly diagnosed CRC is composed of rectal cancer. Currently, the standard treatment for advanced rectal cancer consists of neoadjuvant radiotherapy, with or without sensitizing chemotherapy, followed by surgery with total mesorectal excision (TME) [94,95]. Locally advanced rectal cancer (LARC) is exceptionally challenging to manage, given the structural constrictions of the pelvis. Due to the anatomical challenges encountered during TME, there is an increased risk of operative morbidity and mortality and sexual, urinary, and bowel dysfunction [96].

For smaller tumor lesions and under specific conditions (mostly <4 cm, ≤T3), the neoadjuvant treatment allows avoiding TME for 20–30% of LARC patients who achieve a clinical complete response (cCR) [97,98]. However, the risk of local recurrence and distant metastases remains present within this patient population [99]. Despite improvements in pre-operative care and surgical techniques, the quality of life and survival rates remain subpar among rectal cancer patients. Selecting patients who may most benefit from conservative treatment is crucial.

The clinical application of ctDNA has primarily been evaluated in LARC. At baseline, ctDNA detection could be associated with survival and distant recurrence [100,101,102]. Moreover, ctDNA levels can help monitor the response to radiochemotherapy (RCT) [103]. The detection of ctDNA after radiotherapy [104,105,106,107] or surgery [104,105,107,108,109] is significantly associated with shorter survival (Table 9).

Other studies are currently investigating the significance of ctDNA in directing non-operative management approaches for LARC patients, as shown in Table 10.

## 4. Conclusions and Perspectives

This review provides an up-to-date and comprehensive summary of the various areas of ctDNA research in CRC management. As this review points out, numerous studies have shown the association of ctDNA with tumor burden and its usefulness in detecting and monitoring tumor dynamics, drug response, and resistance to therapy with increasing sensitivities and specificities.

The experimental detection methods are numerous and of great importance in the interpretation of study results. Further improvement in the standardization of methods leading to the preanalytical variability of liquid biopsies is imperative to obtain optimal sensitivity and specificity for the reliable use of ctDNA in daily practice.

At this point, ctDNA detection has yet to be accepted as a worthwhile CRC screening tool. The low concentration available in the early stages imposes the use of highly-sensitive tests, which are currently cost-prohibitive for routine use. The cost of a ctDNA detection assay ranges from EUR 168 to EUR 1423 per sample in a maximum-testing condition, as is expected in future standard practice [111]. Despite these current high costs, active research to improve the methodology and reduce costs is underway. These efforts are driven by the marked advantages of ctDNA, including its accuracy, ease of collection, and minimal invasiveness. It is precisely for this reason that large-scale clinical trials are underway to explore how to optimize ctDNA detection alone or in combination with conventional screening methods.

After surgery, ctDNA can clearly identify patients at low and high risk of relapse, which has direct implications for adjuvant therapy decisions. In the French multicenter adjuvant trial, CIRCULATE-PRODIGE 70, the administration of adjuvant therapy in patients with stage II CRC is based on post-operative ctDNA detection [87]. The ongoing Tracking Mutation in Cell Free Tumor DNA to Predict Relapse in Early Colorectal Cancer (TRACC) study aims to compare ctDNA versus standard of care in predicting relapse in patients with stage II and III CRC undergoing ACT after surgery [90]. In this setting, highly-sensitive tests are critical to avoid both over- and undertreatment.

In patients with metastatic CRC, serial ctDNA testing provides early indications of the clinical efficacy of therapy. In this setting, the variation in ctDNA concentration is related to the response to systemic treatments. Future clinical trials incorporating ctDNA concentration into the study design may allow for the real-time measurement of therapeutic efficacy. Serial testing is also used to validate ctDNA as a detection method of recurrence. One example is the IMPROVE-IT2 (Implementing Noninvasive Circulating Tumor DNA analysis to Optimize the Operative and Post-operative Treatment for Patients with Colorectal Cancer–Intervention Trial 2) trial. This randomized controlled trial investigates the benefit of ctDNA-guided post-operative surveillance compared to the current standard-of-care CT imaging-based surveillance. The main objective is to investigate whether ctDNA-guided surveillance increases the proportion of patients with recurrence receiving curative-intended resection or local metastasis-directed treatment [91].

As data from the many ongoing clinical trials of ctDNA in CRC emerge, better guidelines will arise on incorporating ctDNA into clinical decision-making. Nevertheless, given the heterogeneous nature of colorectal tumors, a single biomarker might be insufficient for managing CRC. Biomarkers could be combined in composite panels such as protein biomarkers, circulating tumor cells, micro RNAs, and ctDNA.

Altogether, evidence strongly indicates that ctDNA should be considered a key tool in the implementation of a personalized medicine approach; it is only a matter of time before ctDNA becomes a crucial part of clinical medicine.

## Figures and Tables

**Table 1:** Comparison of ctDNA detection and analysis methods.

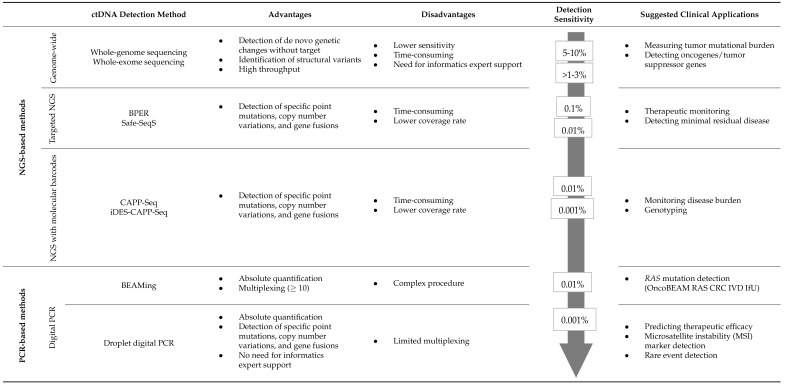

SeqS: Safe-Sequencing System; BPER: Base-Position Error Rate analysis, CAPP-Seq: Cancer Personalized Profiling by deep Sequencing; iDES: integrated Digital Error Suppression; and BEAMing: beads, emulsion, amplification, magnetics.

**Table 2:** Summary of test accuracy results and corresponding (dis)advantages.

	Sensitivity *		Specificity *		Advantages	Limitations
	Adenoma	CRC	Adenoma	CRC		
Colonoscopy	75–95%	18–100%	89–94%	100%	Highly sensitive	Requires sedation Risk for perforations and bleeding
gFOBT	6–17%	50–75%	96–99%	96–98%	Non-invasive	False positives: not fully specific for human hemoglobin
iFOBT/FIT	23%	74%	96%	94%	Non-invasive Specific for human hemoglobin	Restricted specificity considering other bowel diseases may also cause blood in stool
ctDNA (Epi proColon)	22%	68%	79%	79%	Higher compliance than stool-based tests	Low concentrations, limited sensitivity

* Values for sensitivity and specificity were retrieved from the systematic review of Lin et al., containing all relevant studies conducted in asymptomatic populations at general risk of CRC between January 2015 and December 2019 [45].

**Table 3:** Clinical relevance of ctDNA detection at baseline.

	Study	Tumor Stage	Rate of ctDNA Detection before Surgery	ctDNA Detection Method	Outcome
Tumor burden at diagnosis	[52]	I II III	50% 89% 90%	TEC-Seq	Patients with increased pre-operative ctDNA had a shorter PFS and OS compared to patients with a lower ctDNA (HR 1.13, 95% CI: 1.03–1.24)
	[32]		60% 56% 86%	ddPCR	8/10 ctDNA+ patients relapsed 6/11 ctDNA+ patients did not relapse
	[54]		64%	ddPCR	No relation between baseline ctDNA and DFS (HR 0.93, 95% CI: 0.33–2.69)
	[8]		60% 92% 90%	Multiplex PCR-based NGS	No significant association between ctDNA and the outcome
	[55]		30%	ddPCR	Pre-operative ctDNA was associated with inferior RFS (HR 2.18, 95% CI: 1.02–4.61)
	[30]		NM	Spectrophotometry (NanoDrop)	Significantly higher cfDNA levels were observed in patients, with early recurrence compared to non-recurrent patients
	[56]	II III	64%	NGS	Pre-operative ctDNA+ patients had reduced RFS compared with pre-operative ctDNA− patients (HR 5.66; 95% CI: 1.72–18.57)
	[57]		42%	ddPCR	Baseline ctDNA was an independent prognostic factor of DFS (HR 3.35, 95% CI: 1.15–9.77)
	[51]		25% 30%	ddPCR	The rate of recurrence was 32.7% in ctDNA+ patients and 11.6% in ctDNA− patients (*p* = 0.001)
	[53]		64% 74%	Real-time multiplex PCR assay	12/47 (25.5%) ctDNA+ patients relapsed

NGS: Next-Generation Sequencing, TEC-Seq: Targeted Error Correction Sequencing, and NM: not mentioned.

**Table 4:** Clinical relevance of ctDNA as a prognostic marker for the detection of minimal residual disease and recurrence after surgery.

	Study Design	Sample Size	Study Population	ctDNA Detection Method	Timepoint of ctDNA Sampling	Post-Operative ctDNA Detection Rate	Post-Operative Recurrence for ctDNA+ Patients after Surgery	Post-Operative Recurrence for ctDNA− Patients after Surgery
Detection of MRD/recurrence after surgery	Prospective cohort study [64]	230	Stage II CC	Safe-SeqS	4–10 weeks after surgery	8.7%	79%	9.8%
	Prospective cohort study [50]	96	Stage II-III CC	Safe-SeqS	4–10 weeks after surgery	21%	42%	NM
	Prospective cohort study [32]	21	Stage I-III CRC	ddPCR	1–4 weeks after surgery	28.5%	100%	27%
	Cohort study [65]	63	Stage II-III CRC	NGS	Within 1 week after surgery	28.6%	27.8%	4.4%
	Prospective, multi-center cohort study [8]	94	Stage I-III CRC	Multiplex PCR-based NGS (Signatera™)	4 weeks after surgery	10.6%	70%	11.9%
	Prospective study [66]	1017	Stage III CC	ddPCR	35–50 days after surgery	13.8%	After 2 years: 31.4%	After 2 years: 17.2%
	Prospective, multi-center cohort study [51]	184	Stage II-III CRC	ddPCR	1–6 months after surgery	10.5%	44.4%	10.4%
	Prospective, cohort study [56]	240	Stage II-III CRC	425-gene NGS panel-based	3–7 days after surgery	8.3%	60%	NM

CC: colon cancer, Safe-SeqS: Safe-Sequencing System, NM: not mentioned, and NGS: Next-Generation Sequencing.

**Table 5:** Clinical relevance of ctDNA clearance at each stage of treatment.

	Study Design	Sample Size	Study Population	ctDNA Detection Method	Timepoint of ctDNA Sampling	ctDNA Clearance Rate	Outcome
After surgery	Prospective, multi-center cohort study [51]	49	Stage II CRC Stage III CRC	Droplet Digital PCR	Day 5 after surgery	75%	Recurrence rate ctDNA+: 44.4% ctDNA−: 13.7%
	Prospective, multi-center study [56]	240	Stage II CRC Stage III CRC	NGS	Days 3–7 after surgery	92%	2-year RFS: ctDNA+: 39.3% ctDNA−: 89.4%
	Prospective, multi-center cohort study [8]	94	Stage I CRC Stage II CRC Stage III CRC	Multiplex PCR-based NGS	Day 30 after surgery	89%	Recurrence rate: ctDNA+: 70% ctDNA−: 11.9%
After adjuvant chemotherapy	Prospective, multi-center cohort study [8]	10	Stage I CRC Stage II CRC Stage III CRC	Multiplex PCR-based NGS	After completion of chemotherapy	30%	NM
	Prospective cohort study [64]	6	Stage II CC	Safe-SeqS	After completion of chemotherapy	50%	2-year RFS: ctDNA+: 27% ctDNA−: 82%
	Multi-center, cohort study [50]	95	Stage III CC	Safe-SeqS	After completion of chemotherapy	68%	3-year RFI: ctDNA+: 30% ctDNA−: 77%

CC: colon cancer, Safe-SeqS: Safe-Sequencing System, RFS: Regression-Free Survival, NM: Not Mentioned, and RFI: Regression-Free Interval.

**Table 6:** ctDNA in the biological anticipation of radiological recurrence.

	Study Design	Sample Size	Study Population	ctDNA Detection Method	ctDNA Positivity vs. Recurrence Rate	Frequency of Sampling	Delay of Anticipation
**The biological anticipation of radiological recurrence**	Cohort study [68]	58	Stage I–III CRC	Safe-SeqS	ctDNA+ and recurrence: 100% ctDNA− and recurrence: 0%	Post-surgery: 1 month Follow-up: Every 3–6 months	3 months
	Prospective cohort study [32]	27	Stage I–III CRC	ddPCR	ctDNA+ and recurrence: 100% ctDNA− and recurrence: 0%	Post-surgery: Days 8 and 30 Follow-up: Every 3 months	9 months
	Prospective cohort study [64]	178	Stage II CC	Safe-SeqS	ctDNA+ and recurrence: 78.6% ctDNA− and recurrence: 9.8%	Follow-up: Every 3 months	167 days (5 months) (IQR, 81–279 days)
	Prospective study [69]	11	Stage I–IV CRC	ddPCR	ctDNA+ and recurrence: 100% ctDNA− and recurrence: 0%	Post-surgery: Days 8 and 30 Follow-up: Every 3 months	2–15 months (mean of 10 months)
	Prospective, multicenter cohort study [51]	139	Stage II–III CRC	ddPCR	ctDNA+ and recurrence: 32.7% ctDNA− and recurrence: 11.6%	Post-surgery: Day 5 Follow-up: Every 3–6 months	13.1 weeks (IQR, 28 weeks)
	Prospective, multicenter study [70]	160	Stage III CRC	Multiplex PCR-based NGS	ctDNA+ and recurrence: 96% ctDNA− and recurrence: 3%	Follow-up: Every 3 months	9.8 months (IQR, 5–12 months)
	Prospective, multicenter study [56]	276	Stage II–III CRC	NGS	ctDNA+ and recurrence: 76% ctDNA− and recurrence: 4%	Post-surgery: Days 5–8 Follow-up: 6 months after surgery, and then every 3 months	5.01 months

CC: colon cancer, Safe-SeqS: Safe-Sequencing System, ddPCR: droplet digital PCR, and IQR: interquartile range.

**Table 7:** ctDNA as a quantitative monitoring tool in predicting response to treatment.

	Study Design	Sample Size	Study Population	ctDNA Detection Method	PFS
Predicting response to treatment	Prospective, multi-center study [71]	28	Stage IV CRC	ddPCR NGS * (Ion AmpliSeq Cancer Hotspot Panel)	ctDNA−: **4.0 months** ctDNA+: **1.9 months** Hazard ratio, 0.44; 95% CI, 0.18–0.98; *p* = 0.03)
	Clinical trial [72]	29	Stage IV CRC	Guardant 360 ** assay	ApCN ≥ 25.82: **22.5 weeks** ApCN ≤ 25.82: **14.8 weeks** Mantel Cox, *p* = 0.0347
	Prospective study [73]	467	Stage IV CRC	Methy-Light	No effect on PFS
	Prospective study [74]	53	Stage IV CRC	Safe-SeqS	≥10-fold reduction in ctDNA: **14.7 months** ≤10-fold reduction in ctDNA: **8.1 months** Hazard ratio, 1.87; 95% CI, 0.62–5.61; *p* = 0.266
	Prospective (PLACOL) study [61]	82	Stage IV CRC	ddPCR	“good ctDNA responder” = ctDNA concentration < 0.1 ng/mL and SlopeΔctDNA ≥ 80%: **8.5 months** “bad ctDNA responder” = ctDNA concentration > 0.1 ng/mL and SlopeΔctDNA < 80%: **2.4 months** Hazard ratio, 0.19; 95% CI, 0.09–0.40; *p* < 0.0001
	Prospective study [75]	45	Stage IV CRC	dPCR (Methyl-BEAMing)	A negative change in ctDNA is associated with improved PFS

Safe-SeqS: Safe-Sequencing System, ddPCR: droplet digital PCR, and ApCN: adjusted plasma copy number. * Ion Torrent S5 XL, Thermo Fisher Scientific; ** Guardant Health, Inc. Redwood City, CA, USA.

**Table 9:** Studies evaluating the association between ctDNA detection and tumor response in LARC patients.

Study Design	Sample Size	Study Population	ctDNA Detection Method	ctDNA Positivity	Outcome
Cohort study [110]	67	LARC	Real-time PCR	Integrity index before and after CRT of cfDNA	Baseline levels of cfDNA are not associated with tumor response. Post-RCT integrity index is associated with tumor response
Prospective cohort study [103]	4	LARC	NGS	Baseline: 100% After RCT: drop in ctDNA concentration	ctDNA concentration can help monitor response to RCT
Prospective cohort study [104]	159	LARC T3/T4 and/or N+	Safe-SeqS	Baseline: 77% After RCT/before surgery: 8.3% After surgery: 12%	ctDNA+ after RCT and/or surgery is associated with lower 3-year-RFS (33% vs. 87%)
Prospective cohort study [101]	36	LARC	BEAMing	Baseline: 21% After RCT/before surgery: 0%	ctDNA+ at baseline reduced post-operative DFS and OS
Prospective cohort study [105]	47	LARC	ddPCR	Baseline: 74% After RCT initiation: 21% After RCT/before surgery: 21% After surgery: 13%	Patients with ctDNA+ during RCT, after RCT, and after surgery have lower RFS
Cohort study [100]	104	Rectal cancer T4 or N1b-3	NGS	Baseline: 75% 1 month after RCT initiation: 15.6% After RCT/before surgery: 10.5% After surgery: 7.7%	Baseline ctDNA+ is associated with distant recurrence Baseline ctDNA− is associated with pathologic response
Prospective cohort [106]	119	LARC	NGS	Baseline: 86%	ctDNA clearance is associated with tumor regression grade
Prospective study (phase II trial) [102]	71	LARC	NGS	Baseline: 83% After RCT/before surgery: 15%	Pre-operative ctDNA+ is significantly associated with shorter DFS and OS
Prospective cohort study [108]	29	LARC	NGS	Baseline: 65% After RCT/before surgery: 21% After surgery: 13%	Patients with post-operative ctDNA+ experience poor RFS compared to ctDNA− patients
Prospective cohort study [107]	60	LARC	Agnostic and tumor-informed assays	After RCT/before surgery: 23%	ctDNA+ after RCT is associated with lower RFS
Cohort study [109]	51	LARC	NGS	Baseline: 69% After neoadjuvant treatment (chemo + RCT): 15% After surgery: 16%	Patients with post-neoadjuvant treatment and post-operative ctDNA+ experienced poorer RFS than ctDNA− patients

LARC: locally advanced rectal cancer, RCT: radiochemotherapy, DFS: Disease-Free Survival, OS: Overall Survival, and RFS: Recurrence-Free Survival.

**Table 10:** Summary of studies evaluating the utility of ctDNA in LARC patients.

Name of Study and Country	Recruitment Status	Patient Population	Sample Size	ctDNA Detection Method	Intervention	Primary Objective
Circulating Tumor DNA-guided Neoadjuvant Treatment Strategy for Locally Advanced Rectal Cancer (CINTS-R) (NCT05601505) China	Recruiting	Patients with rectal adenocarcinoma who have not received any treatment yet	465	NGS	Control arm: Traditional neoadjuvant chemoradiotherapy Experimental arm: Randomization based on ctDNA results: (1) VAF < 0.4%: neoadjuvant chemoradiotherapy (2) VAF > 0.4%: total neoadjuvant therapy (TNT)	Disease-related treatment failure (DrTF)
Establishing a ctDNA Biomarker to Improve Organ Preserving Strategies in Patients With Rectal Cancer (ctTRAC) (NCT05081024) USA	Recruiting	Patients with stage II–III rectal adenocarcinoma	50	Signatera™ (Natera Inc., Austin, TX, USA)	Observational	Complete clinical response (cCR)
Application of Circulating Tumor DNA Test in the Diagnosis and Treatment of Patients with Advanced Rectal Cancer (NCT03615170) China	Recruiting	Patients with locally advanced rectal cancer, who need to receive neoadjuvant radiotherapy and radical operation	200	Not mentioned	Observational	Disease-free survival
Systemic Neoadjuvant and Adjuvant Control by Precision Medicine in Rectal Cancer (SYNCOPE) (NCT04842006) Finland	Recruiting	Patients with rectal adenocarcinoma that require either radiotherapy or long chemoradiotherapy	93	Not mentioned	Randomization based on ctDNA results: (1) Long-course chemoradiotherapy (2) Total neoadjuvant therapy (TNT): short course RT + capecitabine/oxaliplatin	RFS
